# A pro B cell population forms the apex of the leukemic hierarchy in Hoxa9/Meis1-dependent AML

**DOI:** 10.1038/s41375-022-01775-y

**Published:** 2022-12-14

**Authors:** Anna Lieske, Eric Agyeman-Duah, Anton Selich, Nicole Dörpmund, Steven R. Talbot, Axel Schambach, Tobias Maetzig

**Affiliations:** 1 Institute of Experimental Hematology, Hannover Medical School, Hannover, Germany; 2 Pediatric Hematology and Oncology, Hannover Medical School, Hannover, Germany; 3 Institute for Laboratory Animal Science, Hannover Medical School, Hannover, Germany; 4 Division of Hematology/Oncology, Boston Children’s Hospital, Harvard Medical School, Boston, MA USA

**Keywords:** Cancer stem cells, Acute myeloid leukaemia

## Abstract

Relapse is a major challenge to therapeutic success in acute myeloid leukemia (AML) and can be partly associated with heterogeneous leukemic stem cell (LSC) properties. In the murine *Hoxa9/Meis1*-dependent (H9M) AML model, LSC potential lies in three defined immunophenotypes, including Lin^−^cKit^+^ progenitor cells (Lin^−^), Gr1^+^CD11b^+^cKit^+^ myeloid cells, and lymphoid cells (Lym^+^). Previous reports demonstrated their interconversion and distinct drug sensitivities. In contrast, we here show that H9M AML is hierarchically organized. We, therefore, tracked the developmental potential of LSC phenotypes. This unexpectedly revealed a substantial fraction of Lin^−^ LSCs that failed to regenerate Lym^+^ LSCs, and that harbored reduced leukemogenic potential. However, Lin^−^ LSCs capable of producing Lym^+^ LSCs as well as Lym^+^ LSCs triggered rapid disease development suggestive of their high relapse-driving potential. Transcriptional analyses revealed that B lymphoid master regulators, including *Sox4* and *Bach2*, correlated with Lym^+^ LSC development and presumably aggressive disease. Lentiviral overexpression of *Sox4* and *Bach2* induced dedifferentiation of H9M cells towards a lineage-negative state in vitro as the first step of lineage conversion. This work suggests that the potency to initiate a partial B lymphoid primed transcriptional program as present in infant AML correlates with aggressive disease and governs the H9M LSC hierarchy.

## Introduction

Acute myeloid leukemia (AML) represents a hematopoietic disorder characterized by the accumulation of immature blast cells and poor overall survival rates. Like normal hematopoiesis, AML is hierarchically organized with the leukemic stem cell (LSC) at its apex, which harbor self-renewal potential and thus initiate and maintain the disease. Therapeutic elimination of LSC is hampered by their mutational, epigenetic, transcriptional, metabolic, and phenotypic heterogeneity [1]. Thus, differential LSC characteristics form the root of diverse treatment responses and prognoses [2–4]. Consequently, strategies to better understand the clonal heterogeneity of LSC at different stages of disease progression are urgently needed to develop improved therapeutic regimens.

A particular form of LSC heterogeneity is represented by phenotypic plasticity and describes the potency of LSC subpopulations to adapt and to merge into different phenotypes, e.g., the absence or presence of markers characteristic of single or ambiguous lineage-identity [5]. This represents a hallmark of mixed lineage leukemia (MLL) characterized by translocations of the *Lysine Methyltransferase 2A* (*KMT2A*, aka *MLL*) gene [6, 7]. Consequently, the MLL-fusion protein stabilizes the expression of the homeoboxproteins HOXA9 and MEIS1, which are frequently overexpressed in poor prognosis AML and contribute to malignant transformation [8]. Notably, MLL-translocations account for >15% of AML in young patients as opposed to <3% in adults [9].

Human AML with rearranged MLL can be modeled in mice through the direct overexpression of *Hoxa9* and *Meis1* (H9M) in murine bone marrow cells [5]. Gibbs et al. showed that H9M LSC reside in three immunophenotypically distinct cell populations: Lin^−^cKit^+^ progenitor cells (Lin^−^), Gr1^+^CD11b^+^cKit^+^ myeloid cells (Myeloid) and Lym^+^ (CD3^+^ and/or B220^+^) cKit^+^ lymphoid cells (Lym^+^) [5]. In transplantation assays, these LSC immunophenotypes reconstituted each other but displayed distinct drug sensitivities [5], which links phenotypic plasticity with relapse. However, the underlying molecular pathways remained elusive. In contrast to the described phenotypic plasticity of all H9M LSC subpopulations, our previous work uncovered a reduced frequency of animals engrafted with a Lym^+^ LSC subpopulation [10]. This suggests a hierarchical organization of the LSC pool, where dedifferentiation towards Lym^+^ LSCs does not proceed by default. Conceivably, alterations in lineage identity depend on specific gene-regulatory networks. For example, in normal hematopoiesis, the establishment of a B lymphoid fate relies on the expression of the transcriptional repressor *Bach2* in common lymphoid progenitors (CLP) [11, 12], and the subsequent activation of *Ebf1* and *Pax5* for final B lineage commitment [13]. In this process, the survival of pro B cells is mediated by *Sox4* [14], which, similar to *Bach2*, impairs C/EBP-driven myeloid differentiation.

Notably, we detected the lymphoid differentiation block in H9M-driven AML through the use of a fluorescent genetic barcoding (FGB) approach [10]. FGB depends on the individual lentiviral labeling of bulk populations with different fluorescent protein combinations, the mixing of these cells and their subsequent tracking by flow cytometric analyses. In contrast to conventional transplantation assays with a single (e.g., GFP) labeled population, FGB enables the longitudinal characterization of multiple color-coded populations in parallel along with the opportunity to assess population-specific differentiation capacities and phenotypes. In comparison to DNA barcode-based tracking approaches [15–17], FGB thus benefits from the opportunity to identify and isolate viable cells of interest for subsequent studies by flow cytometric techniques as demonstrated for LSC and hematopoietic stem cells (HSC), respectively [10, 18, 19].

We opted to further investigate the phenotypic plasticity and leukemic hierarchy in the H9M AML model by utilizing a combination of FGB-mediated multiplex transplantation assays, serial transplantation of defined LSC immunophenotypes, and their characterization by transcriptional analyses and functional assays. These experiments revealed a pro B cell population at the apex of the leukemic hierarchy, and linked phenotypic plasticity with aggressive disease and relapse potential that might also play a role in human AML.

## Material and methods

Experimental procedures are detailed in the Supplementary Materials and Methods section.

## Results

### Generation of a resource bank of color-coded H9M LSC samples

**Fig. 1:**
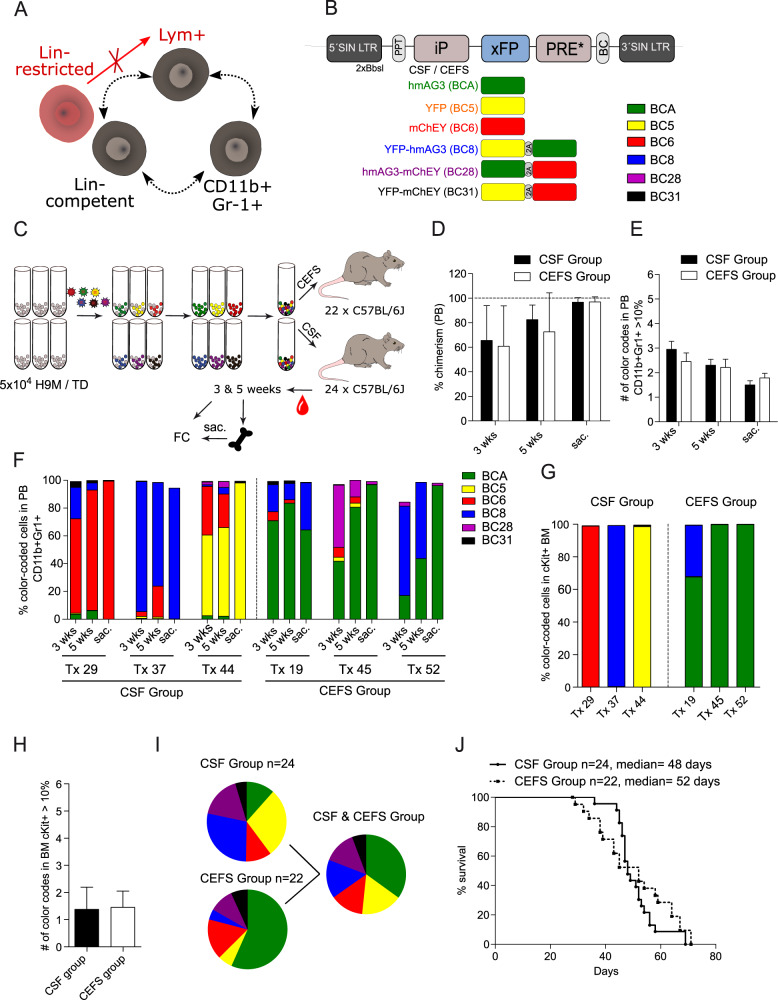
The 6xFGB vector platform facilitates in vivo multiplexing of H9M cells. **A** Hypothetical connection of three different LSC subpopulations characterized by their ability to regenerate each other. **B** The 6xFGB vector system consists of 6 different vectors expressing unique color codes (hmAG3, YFP, mChEY, YFP-hmAG3, hmAG3-mChEY, and YFP-mChEY) linked to specific DNA barcodes (BC) for identification. Transgene expression is driven by the CSF or CEFS promoter. **C** Experimental design. The 6 FGB vectors (with CSF or CEFS promoter) were individually transduced into separate H9M cultures. After expansion, pure color-coded cells were sorted and mixed in equal ratios for transplantation into lethally irradiated mice. Flow cytometry enabled the longitudinal color code tracking in peripheral blood (PB) samples as well as in end-point bone marrow (BM) samples. **D** PB chimerism over time for the CSF and CEFS group based on the CD45.1^+^ donor population 3 and 5 weeks post-transplantation and at the time of sacrifice. **E** The number of color codes with contributions >10% to the donor-derived PB myeloid CD11b^+^Gr1^+^ fraction over time. **F** Exemplified real-time tracking of color codes in the PB. Cells were first gated on the CD45.1^+^CD11b^+^Gr1^+^ fraction before calculating the size of each color code as a percentage of all color-coded cells within the population. **G** Exemplified color code distribution in the BM within the CD45.1^+^-derived cKit^+^ population. The size of each color code was expressed as a percentage of all color-coded cells within the population. **H** The number of color codes with contributions >10% to the donor-derived cKit^+^ BM fraction at the end of the experiment. **I** Frequency of color codes in the CD45.1^+^-derived cKit^+^ BM population of mice transplanted with CSF-derived H9M cells (*n* = 24), CEFS-derived H9M cells (*n* = 22), and of the combined dataset. **J** Kaplan–Meier survival curves of mice transplanted with the 6xFGB H9M cells. **D**, **E**, and **H** show the mean  ± SD from 22 (CEFS group) and 24 (CSF group) transplanted mice, respectively. BC barcode, FC flow cytometry, FGB fluorescent genetic barcoding, iP internal promoter, PPT polypurine tract, PRE posttranscriptional regulatory element, SIN LTR self-inactivating long terminal repeat, xFP fluorescent protein cassette, SD standard deviation, hmAG3 modified humanized Azami Green, YFP yellow fluorescent protein, mChEY modified mCherry.

To isolate LSC subpopulations with differential lineage plasticity for functional studies, we opted to recapitulate our previous multiplexing experiments with an improved 6xFGB vector system (Fig. 1A, B, C) [10, 19]. These vectors were equipped with silencing-resistant versions of a physiologic elongation factor 1 alpha short (CEFS) promoter and a spleen focus-forming virus (CSF) promoter, respectively, and allowed for reliable multiplex tracking in vitro (Fig. S1). The advantage of our multiplexing approach lies in the comparability of phenotypes between the six color-coded input populations. Thus, the presence of a dedicated color code in all LSC subpopulations would report on “Lin^−^ competent” and “Lym^+^ competent” cells with the capacity to reconstitute all immunophenotypes. In contrast, the absence of a color code in any given subpopulation would hint towards a “lineage-restricted” Lin^−^ progenitor cell population, which produces itself but fails to regenerate lymphoid LSCs (Fig. 1A red).

To monitor a diverse and representative set of color-coded populations despite the expected clonal restriction caused by cell intrinsic inheritable features [20], we transplanted a total of 24 and 22 animals with color-coded H9M cells transduced with the CSF- and CEFS-derived 6xFGB vector series, respectively, in three independent experiments. These in vivo competition assays of 6xFGB color-coded H9M cells were initiated with 1 x 10^5^ cells per color code, which, based on previous limiting dilution experiments with independently generated H9M lines, are expected to contain >50 LSC, allow for uniform engraftment and cause end-stage disease with a median of 40 to 60 days [10]. Mice from both FGB-labeling groups showed a comparable peripheral blood (PB) chimerism over time with nearly 100% penetrance at the leukemic end-point and elevated white blood counts and spleen weights representative of full-blown leukemic burden (Fig. 1D and Fig. S2). As expected from leukemic selection, the number of color codes with contributions >10% to the myeloid fraction of the PB decreased over time starting from 3.0 ± 1.6 and 2.5 ± 1.6 at week 3 and declined to 1.5 ± 0.8 and 1.8 ± 0.8 at time of sacrifice for the CSF and CEFS groups, respectively (exemplified gating strategy in Fig. S3) (Fig. 1E). At the leukemic end-point, the color code distribution in the PB matched with those of the LSC entity of the cKit^+^ BM fraction (Fig. 1F–I; exemplified gating strategy for BM in Fig. S4). Furthermore, the median survival of all mice was 48.5 days with no significant differences induced by the different color-coding vector series (CSF group 48 days vs. CEFS group 52 days) (Fig. 1J), and reflected the typical survival periods for the H9M model described in the literature [21–23].

In summary, our 6xFGB vector platform allowed for the competitive in vivo real-time tracking of color-coded H9M populations. Since both vector configurations (CEFS and CSF) comparably recapitulated leukemogenesis, subsequent experiments with the banked cell material did not distinguish between the labeling constructs anymore.

### In vivo multiplexing of H9M cells revealed the aberrant appearance of immunophenotypically distinct LSC subpopulations

**Fig. 2:**
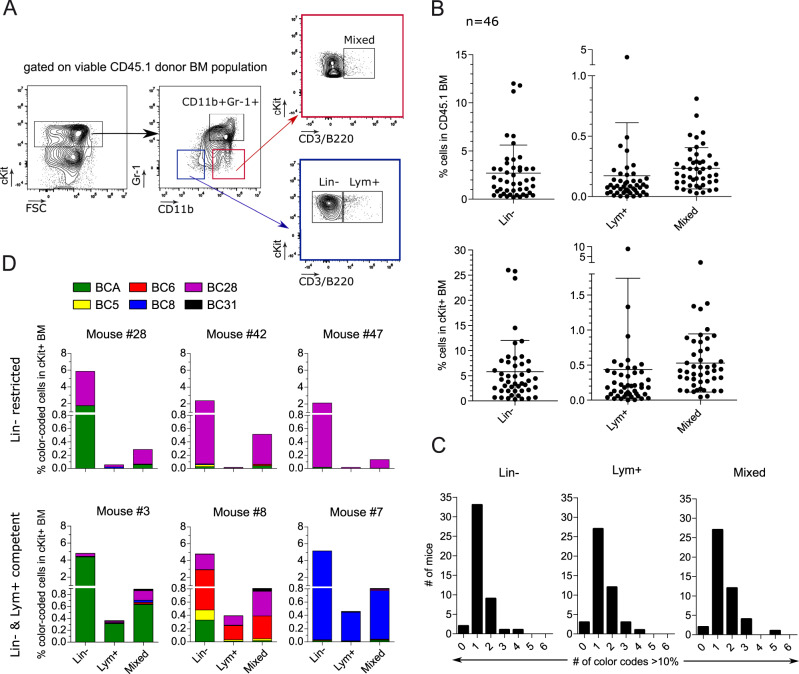
Color-coded H9M cells differentiate into immunophenotypically diverse BM LSC populations. **A** Exemplified gating strategy for the identification of H9M LSC immunophenotypes in the BM. Viable donor-derived CD45.1^+^ cells were first gated on the cKit^+^ marker. The myeloid marker combination CD11b^+^ and Gr1^+^ revealed double-positive myeloid LSCs as a first subpopulation. Furthermore, Lin^−^ progenitor cells and lymphoid CD3/B220^+^ cells were identified as second and third populations by gating on cKit and CD3/B220. A fourth population, named Mixed, was discovered by gating on CD11b^+^ and CD3/B220^+^ cells. **B** Population sizes of the LSC immunophenotypes in the BM. The percentages of Lin^−^, Lym^+^ and Mixed cells within the parental CD45.1^+^ and cKit^+^ BM populations for all 46 recipients. Each dot represents one mouse. Mean  ± SD is shown. **C** Number of mice containing 0 to 6 different color codes for each immunophenotype. Cells were first gated on the three immunophenotypes before assessing the color code distribution and counting the number of color codes with contributions >10% of all color-coded cells in the indicated (Lin^−^, Lym^+^, and Mixed) BM LSC subpopulations. The analysis included all 46 recipients. **D** Exemplified contributions of color-coded Lin^−^, Lym^+,^ and Mixed LSC populations to the cKit^+^ BM of 6 representative mice. Animals were grouped according to a Lin^−^ restricted or Lin^−^ and Lym^+^ competent phenotype.

We next aimed to reveal disparities of the color code distribution between the Lin^−^ and Lym^+^ subpopulations that would suggest an organized LSC hierarchy in the BM. We, therefore, employed flow cytometry to assess the distribution of the previously described LSC immunophenotypes in end-point BM samples by gating on the LSC-enriched cKit^+^CD45.1^+^ donor cell fraction and on myeloid (Gr1 and CD11b) and lymphoid (CD3 and B220) markers therein (Fig. 2A) [5, 24]. These analyses yielded an unexpected “Mixed” population characterized by the co-expression of myeloid and lymphoid markers (cKit^+^CD11b^+^CD3/B220^+^). The frequency of Lin^−^ LSC, Lym^+^ LSC, and Mixed cells in the CD45.1^+^ population accounted for 2.7 ± 2.9%, 0.2 ± 0.4% and 0.2 ± 0.2%, respectively (Fig. 2B). The subsequent evaluation of the color code distribution revealed the predominant expression of the same color code in all three LSC subpopulations per mouse (Fig. 2C and Fig. S5 generated according to the gating strategy depicted in Fig. S4), as exemplified for BCA in Mouse #3 and BC8 in Mouse #7 (Fig. 2D). However, we observed 12 animals with a completely absent or vanishingly small (0.0 to 0.02% in cKit) color-coded Lym^+^ population. For instance, the BCA color code was present in the Lin^−^ and Mixed populations with 1.73% and 0.06% of the cKit^+^ fraction, but absent in the Lym^+^ phenotype in Mouse #28. In 5 recipients (10.9%), even the whole Lym^+^ population was missing (e.g., Mouse #42 and Mouse #47; Fig. 2D). Moreover, some animals (e.g., Mouse #8), suggested the coexistence of Lin^−^ competent (BC6 and BC28) and Lin^−^ restricted (BCA and BC5) color codes.

Together, the disparate color code expression between the Lin^−^ and Lym^+^ LSC subpopulations, as well as the occasional complete absence of the Lym^+^ LSC subpopulation point towards a directional regulation of phenotypic LSC plasticity.

### Secondary transplantations prove the hierarchical organization of the H9M LSC niche

**Fig. 3:**
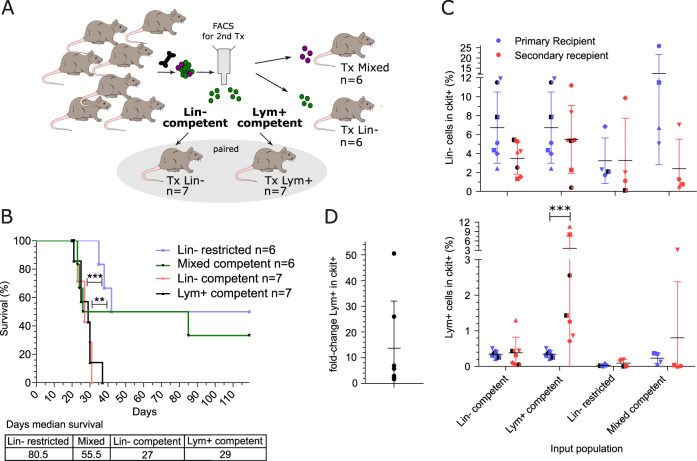
In vivo tumorigenic differentiation proceeds along a hierarchical succession. **A** Experimental setup of secondary transplantations. Four different LSC phenotypes were sorted from primary BM (1 x 10^3^ cells per recipient) for serial transplantation: (I) Lin^−^ restricted LSCs, (II) Mixed competent LSCs, (III) Lin^−^ competent LSCs and (IV) Lym^+^ competent LSCs. Notably, similar paired color-coded Lin^−^ and Lym^+^ competent LSCs were isolated from the same donors, while Lin^−^ restricted LSCs and Mixed competent LSCs were independently purified. **B** Kaplan–Meier survival curves and log-rank test of secondary transplanted mice. 2 of the Lin^−^ restricted mice did not engraft (BM chimerism <1%, 4 months after transplantation), and one additional animal had not reached the leukemic end-point at the time of mandatory sacrifice 4 months after transplantation. Additionally, 2 of the mice from the Mixed group did not engraft. **C** Size comparison of BM LSC immunophenotypes between the primary donor animals (blue) and the secondary recipients (red). Each point represents one animal. Statistical differences were investigated by Mann–Whitney *U* test. **D** Fold-change of BM Lym^+^ LSC between the primary and secondary recipients. Mean ± SD is shown. **p ≤ 0.01, ***p ≤ 0.001.

To functionally verify the unidirectional hierarchical organization of H9M AML and to exclude the possibility of delayed Lym^+^ cell formation in primary hosts, we next performed serial transplantation experiments of sorting-enriched color-coded LSC subpopulations. For these experiments, we co-purified Lin^−^ competent and Lym^+^ competent LSCs from the same hosts (*n* = 7) for direct correlation of their differentiation preferences as well as Lin^−^ restricted LSC and the newly identified Mixed population from independent donors (Fig. 3A and Table S1). Here, the main criterion for the selection of donor mice was the presence of a sufficiently large Lym^+^-competent LSC population that would allow its enrichment by cell sorting. Interestingly, we observed the longest median survival for the Lin^−^ restricted group (80.5 days) as well as for the newly identified Mixed LSC subpopulation (55.5 days). In contrast, all recipients receiving Lin^−^ (*n* = 7) and Lym^+^ (*n* = 7) competent LSCs developed aggressive AML with a median survival of 27 and 29 days, respectively, which was significantly reduced compared to the Lin^−^ restricted group (Fig. 3B).

To further assess the differentiation potential of each purified LSC subpopulation, we determined the frequency of Lin^−^cKit^+^ and Lym^+^cKit^+^ cells in the BM for each secondary recipient compared to the respective primary donor. Similar to primary AML, all LSC subpopulations produced Lin^−^ cells whereby Lin^−^ competent cells indicated reduced self-renewal potential with only 3.5 ± 1.7% in the cKit^+^ BM fraction compared to primary donor mice (6.7 ± 3.7%) (Fig. 3C upper graph). Importantly and similarly to primary recipients, the transplantation of Lin^−^ restricted LSCs failed to produce Lym^+^ cells, which confirmed their impaired regenerative potential (Fig. 3C lower graph). Strikingly, we detected a significant increase in Lym^+^ LSC in all secondary animals transplanted with Lym^+^ competent donor cells (0.3 ± 0.1% in primary AML vs. 3.6 ± 4.0% in secondary AML), which was on average 13.7 ± 18.3-fold higher in secondary recipients, indicating their enriched self-renewal potential (Fig. 3D).

These results suggest that the ability to produce lymphoid cells is indicative of increased LSC content and their localization at the top of the leukemic hierarchy.

### Single-cell RNA sequencing locates pro B cells at the apex of the LSC hierarchy

**Fig. 4:**
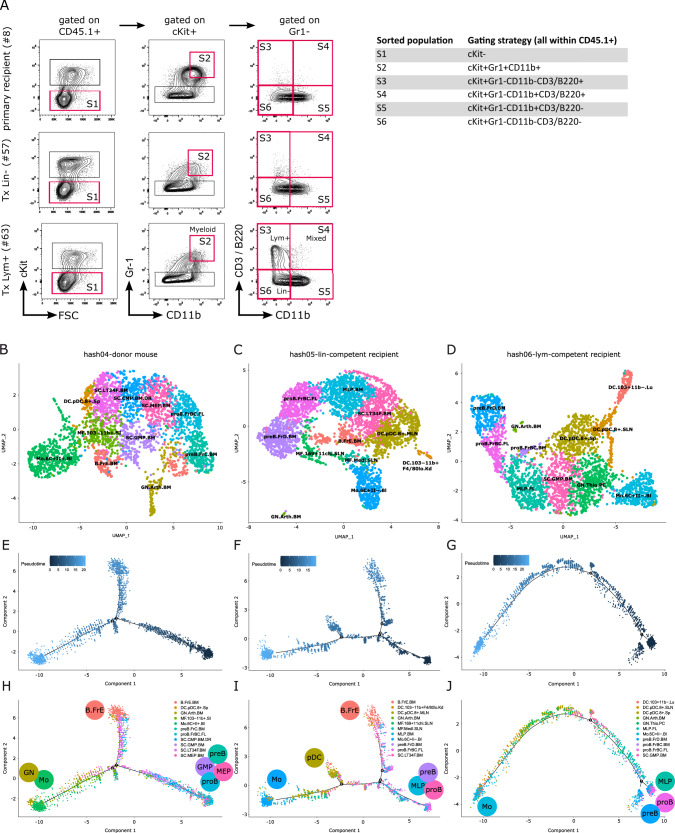
ScRNA-seq reveals the differentiation trajectory of H9M AML in vivo. **A** Gating strategy for the enrichment of scRNA-seq dedicated H9M populations from a donor mouse (#8), and its paired secondary recipients of Lin^−^ competent cells (mouse #57), and Lym^+^ competent cells (mouse #63). Sorted target populations (S1–S6; 1 x 10^3^ cells each) are indicated. **B**–**D** Cell type composition of the three scRNA-seq samples after hash-tag deconvolution as determined by CIPR using the ImmGen reference dataset. **E**–**G** Pseudotime trajectory and **H**–**J** overlaid cell populations for investigation of differentiation processes underlying the H9M hierarchy. ImmGen reference cell type abbreviations: SC.LT34F.BM, Long-term reconstituting stem cell; SC.MEP.BM, Megakaryocyte-Erythroid Progenitor; Mo.6C+II+.Bl, Classical Monocytes, MHCII+; proB.FrBC.FL, Fr. B/C (Pro-B); SC.CMP.BM.DR, Common Myeloid Progenitor; MF.103-11b+.SI, Small Intestine Lamina Propria Cd103- Cd11b+ Dendritic Cells; preB.FrC.BM, Fr. Cprime (Cycling Pre-B); SC.GMP.BM, Granulocyte-Monocyte Progenitor; B.FrE.BM, Newly-formed B Cell Population, Fr. E; GN.Arth.BM, Neutrophils, Arthritic; DC.pDC.8+.Sp, Spleen CD8+ Plasmacytoid Dendritic Cell; MLP.BM, Multilineage Progenitor; preB.FrD.BM, Small pre-B Population, Fr. D; DC.pDC.8+.MLN, Mesenteric LN Cd8+ Plasmacytoid Dendritic Cell; MF.169+11chi.SLN;Lymph Node Cd169+ Cd11C+ MF (Cd169+ DC); MF.Medl.SLN, Medullary Sinus MF (Mm); DC.103-11b+F4/80lo.Kd, Kidney Cd11b+ F4/80Lo Dendritic Cells (P7, Cd11b Hi); GN.Thio.PC, Neutrophils, Thioglycolate; MLP.FL, Multilineage Progenitor; Mo.6C+II-.Bl, Classical Monocytes, MHCII-; DC.pDC.8+.SLN, Skin Draining LN Cd8+ Plasmacytoid Dendritic Cell; DC.103+11b-.Lu,Lung CD103+ Dendritic Cells; proB.FrBC.BM, pro-B Stage Cell Population, Fr. B & Fr. C.

We next wondered if the hierarchical organization of the LSC subpopulations would also be reflected in their transcriptomes. For this analysis, we selected a set of three BM samples derived from a primary donor mouse (mouse #8) and its two recipients of Lin^−^ competent LSC (mouse #57) and Lym^+^ competent LSC (mouse #63), respectively. This group of mice was chosen for single-cell RNA-sequencing (scRNA-seq) based on their shared cell material and the strong expansion of the Lym^+^ LSC population in Mouse #63, which facilitated the purification of sufficient cell numbers by FACS. From each mouse, 1 x 10^3^ hash-tagged cells from each of the 6 phenotypically-defined BM subpopulations were subjected to scRNA-seq (Fig. 4A) [25]. Hash-tag-specific clustering was followed by automated cell type annotation by CIPR (Tables S2–S5) [26]. These analyses showed that the donor mouse (#8; hash 04) contained various immature cell populations including myeloid progenitors (granulocyte monocyte progenitor (GMP), common myeloid progenitor (CMP), and megakaryocyte erythrocyte progenitor (MEP)) and long-term HSC. Moreover, differentiated cell populations, such as plasmacytoid dendritic cells (pDC), macrophages, monocytes, and granulocytes emerged. In line with the original detection of a B220^+^ cell population in H9M BM, CIPR also annotated multiple clusters as B progenitors, including an immature proB FrBC cluster (Fig. 4B).

The recipient mouse of Lin^−^ competent cells (#57; hash 05) still contained long-term HSC, mature myeloid cell populations as well as B progenitors including a proB FrBC cluster (Fig. 4C). However, instead of the myeloid progenitor compartment, a multi-lymphoid progenitor (MLP) cluster appeared. This cluster was also present in the recipient mouse of Lym^+^ LSC (#63; hash 06) and was accompanied by a GMP cluster. Again, various B cell clusters were identified, including the proB FrBC cluster (Fig. 4D).

Since these mice shared the proB FrBC cluster, but presented with unique sets of known progenitor populations, we next wondered about the differentiation trajectories of the different cell types (Fig. 4E–J). In all mice, the proB cell population clustered together with other progenitors (GMP and MEP in mouse #8, and MLP in mice #57 and #63), which were placed at the beginning of the pseudotime trajectory just opposite to differentiated monocytes. Therefore, secondary transplantation of Lin^−^ competent cells as well as Lym^+^ competent cells enrich for lymphoid progenitor populations at the apex of the differentiation trajectory, including an immature proB FrBC cell population.

### Gene expression profiling identified the upregulation of lymphoid driver genes in the Lym^+^ LSC compartment

**Fig. 5:**
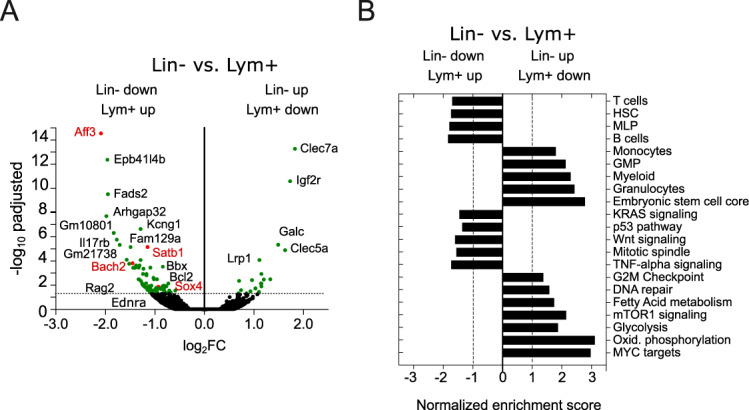
Comparative bulk RNA-sequencing identified genes important for lymphoid lineage specification. **A** The volcano plot comparably shows the global gene expression of Lym^+^ and Lin^−^ LSCs. A Log_2_FC < 0 and Log_2_FC > 0 was chosen as detection limit for upregulated and downregulated genes, respectively. Green dots represent genes significantly deregulated between Lym^+^ and Lin^−^ LSCs, and red dots represent potential drivers of lymphoid lineage identity (P-adj < 0.05). **B** Gene set enrichment analysis.

To uncover the molecular mechanisms governing the plasticity and self-renewal potential of H9M LSC, we aimed to compare the global gene expression profiles of sorted Lym^+^ and Lin^−^ *competent* cells derived from three independent secondary recipients, each. This identified the significant upregulation of lymphoid-primed transcription factors *Aff3* (AF4/FMR2 family member 3), *Bach2* (BTB domain and CNC homology 2), *Satb1* (special AT-rich sequence-binding protein-1), and *Sox4* (SRY-box transcriptions factor 4) in the Lym^+^ compartment (Fig. 5A left). All of which play essential roles during lymphoid lineage development [11, 14, 27–29]. Furthermore, gene set enrichment analyses (GSEA) identified differentially regulated pathways between Lin^−^ and Lym^+^ LSCs. By using gene sets related to hematopoiesis [30], we verified for Lym^+^ cells lymphoid pathways dedicated to late lymphoid differentiation, and B and T cells. In addition, gene sets associated with HSC were upregulated in comparison to the Lin^−^ fraction. In contrast, pathways dedicated to myeloid cells were enriched in Lin^−^ LSCs (Fig. 5B).

By assessing differentially regulated pathways between Lym^+^ and Lin^−^ LSCs, an upregulation of Kras, p53, and Wnt signaling was verified in Lym^+^ LSCs in contrast to metabolic pathways, Myc and mTOR signaling in Lin^−^ cells (Fig. 5B) [31]. The protein-protein interaction network of differentially expressed genes in the Lym^+^ LSC compartment revealed, furthermore, upregulation of genes involved in NOTCH signaling, including *Maml2* (P-adj 0.04), *Maml3* (P-adj 0.07), and *Jag2* (P-adj 0.08) that converge on *Crebbp* (P-adj 0.09) as a central node, although most of these genes failed to show significant deregulation between the two groups (Fig. S6) [32].

In summary, bulk RNA-sequencing verified the overexpression of genes involved in lymphoid lineage development in Lym^+^ LSCs, which are furthermore enriched in gene sets associated with increased leukemic potential [3, 33–35].

### The overexpression of lymphoid driver genes leads to a context-dependent growth advantage of H9M cells

**Fig. 6:**
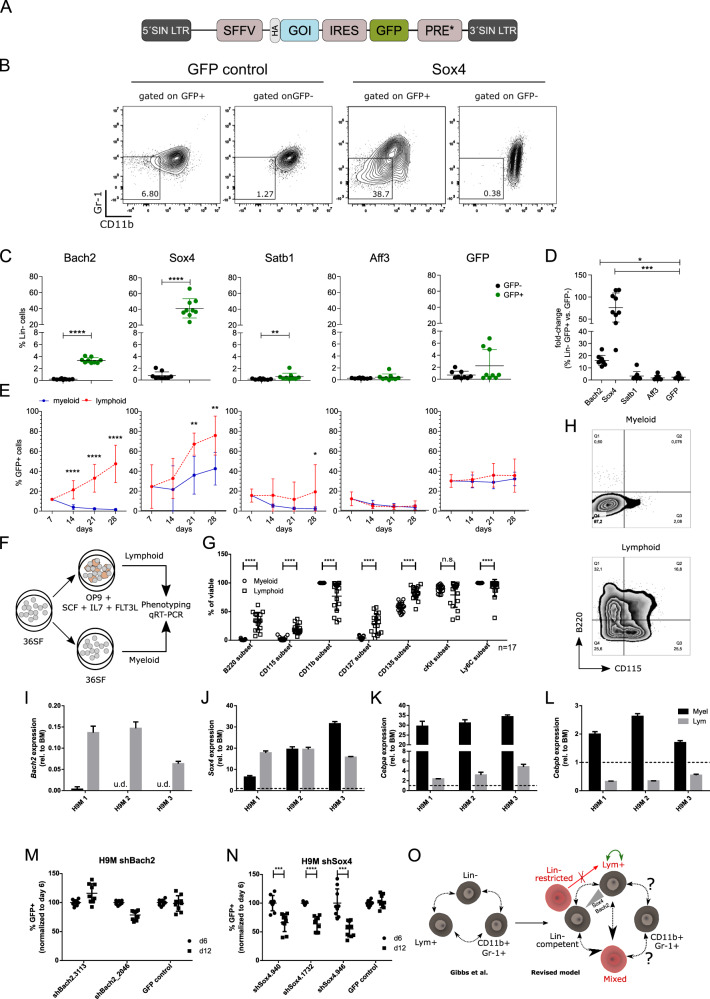
Overexpression of lymphoid transcription factors and modified culture conditions of H9M cells cause loss of myeloid lineage specification in vitro. **A** Lentiviral vector design for the overexpression of candidate gene cDNAs. The lentiviral vector consists of the gene of interest (GOI), an SFFV promoter, an IRES and a GFP cassette to track transduced cells. **B** Exemplified gating strategy for the identification of Lin^−^ cells in the GFP^+^ and GFP^−^ fraction of the GFP control and *Sox4* transduced H9M cells after 7 days. **C** Percentage of Lin^−^ (CD11b^−^, Gr-1^−^) cells in the cKit^+^GFP^+^ and cKit^+^GFP^−^ population determined 7 days after transduction. Statistical differences were determined by Mann–Whitney *U* test. **D** Fold change of the percentage of Lin^−^ cells between the cKit^+^GFP^+^ and cKit^+^GFP^−^ populations for each of the indicated cDNAs and compared to the GFP control sample. Statistical differences were determined by Kruskal–Wallis test with Dunn’s multiple comparisons test. **E** Longitudinal flow cytometric analysis of transduced cells. Cells were independently transduced with the GFP control vector or vectors carrying a cDNA for *Bach2*, *Satb1*, *Sox4* or *Aff3* under myeloid cultivation conditions. 7 days after transduction, cells were first measured by flow cytometry before being further cultivated under myeloid conditions (blue) or transferred into lymphoid cultivation conditions together with OP9 cells (red). GFP signals were measured every 7 days for 28 days. Statistical differences were determined by Mann–Whitney *U* test. Data in **C**–**E** represent mean values  ± SD from three independent experiments (each *n* = 3). **F** GFP transduced H9M cells were subjected to extended in vitro cultivation in standard myeloid-inducing cytokine conditions (36SF) or on OP9 feeder cells in the presence of B cell-inducing cytokines (mSCF, hIL7 and hFLT3L) for phenotyping and gene expression analysis. **G** Phenotyping of three different parental H9M lines split into 17 wells for 45 days of exposure to lymphoid culture conditions. Myeloid cells were maintained and analyzed in parallel. Statistical differences were determined by Mann–Whitney *U* test. **H** Representative distribution of B220 and CD115 (CSF1R) expression on H9M cells cultivated in myeloid or lymphoid conditions. **I**–**L** Quantification of mRNA expression for three independent H9M lines cultivated under myeloid and lymphoid conditions, respectively. Expression differences were assessed for (**I**) *Bach2*, (**J**) *Sox4*, (**K**) *Cebpa*, and (**L**) *Cebpb,* and expressed relative to a normal bone marrow (rel. BM) control, which was set to “1” (dotted line). **M**, **N** shRNA knockdown assays for suppression of (**M**) *Bach2* and (**N**) *Sox4*. 3 different H9M lines were transduced in triplicates with indicated shRNAs (GFP^+^) as well as a GFP control vector (shared at day 6 between *Bach2* and *Sox4* assays). GFP marking rates were determined by flow cytometry 6 and 12 days post transduction. Statistical differences were determined by paired two-sided *t* test. **O** Comparison of the H9M AML model of Gibbs et al. (left), which assumes phenotypic plasticity of all LSC subpopulations [5], with a revised hierarchical system based on our work (right). *p ≤ 0.05, **p ≤ 0.01, ***p ≤ 0.001 and ****p ≤ 0.0001. u.d. undetermined/no signal.

We next hypothesized that the overexpression of lymphoid-primed transcription factors in H9M cells would lead to increased self-renewal potential. Seven days after transduction with lentiviral vectors coexpressing our candidate genes (*Bach2, Satb1, Sox4, and Aff3*) and GFP (Fig. 6A), H9M cells were analyzed for the expression of cKit, Gr-1, CD11b, CD3, and B220 by flow cytometry (Fig. 6B). All transduced GFP^+^ cells maintained their cKit^+^ marker expression, similar to untransduced GFP^−^ cells within the same cultures (data not shown). Interestingly, *Bach2* and *Sox4* expression led to the downregulation of myeloid markers and the acquisition of an immature Lin^−^cKit^+^ phenotype (Fig. 6C, D). Thereafter, aliquots of the cells were kept in standard myeloid culture conditions or transferred into B lymphoid media conditions to investigate the growth behavior under the influence of lineage-instructive cytokines and stroma. Here, *Bach2* overexpression led to a 20.6 ± 2.5-fold (at day 28) expansion under lymphoid compared to myeloid cultivation conditions. In addition, the transduction of *Sox4* facilitated a strong expansion of H9M cells independent from the cultivation conditions (Fig. 6E).

### Induction of phenotypic plasticity through altered culture conditions

Given the growth-promoting function of *Bach2* in lymphoid conditions, we wondered to what extent the cultivation conditions alone would influence H9M characteristics (Fig. 6F), and subjected long-term cultures (45 days) of GFP transduced H9M cells to phenotyping. Surprisingly, this revealed the upregulation of B220 expression and the downregulation of CD11b expression compared to myeloid cultures (Fig. 6G). Moreover, lymphoid-related markers such as IL7R (CD127) and FLT3 (CD135) were upregulated and presumably replaced cKit function. Regardless, CD115 showed the most heterogeneous expression profile (Fig. 6G, H), which typically marks cells of the mononuclear phagocyte system consisting of progenitors, monocytes, macrophages, and classical dendritic cells [36]. In line with the adaptation of a lymphoid primed phenotype, these cells showed the upregulation of *Bach2* by RT-qPCR, along with the downregulation of myeloid-instructive *Cebpa* and *Cebpb*, and the relatively stable expression of *Sox4* between myeloid and lymphoid conditions (Fig. 6I–L).

In summary, the overexpression of *Bach2* and *Sox4* leads to culture-independent (*Sox4*) and lymphoid-specific (*Bach2*) H9M expansion. However, the induction of phenotypic plasticity seems to depend on the long-lasting stimulation of cytokine and/or stroma-dependent mechanisms.

### *Sox4* knockdown leads to rapid H9M depletion

We next investigated, if *Sox4* and *Bach2* are essential for H9M cell growth. For these experiments, shRNAs were validated by transient reporter assays and typically achieved reporter depletion to 20% of the control (Fig. S7A, B). While *Bach2*-targeted shRNAs did not influence H9M growth presumably due to very low or absent expression in H9M cells under myeloid instructive cytokines (Fig. 6I, M), *Sox4* suppression led to rapid cell depletion in three different H9M lines (Fig. 6N).

These data suggest a model, in which the combined action of *Bach2* and *Sox4* contribute to phenotypic plasticity (*Bach2*) and increased aggressiveness *(Sox4*) in a fraction of susceptible LSC that subsequently become Lym^+^ LSC and locate at the top of the leukemic hierarchy (Fig. 6O).

### *BACH2* is upregulated in infant AML with a B lymphoid transcription program

**Fig. 7:**
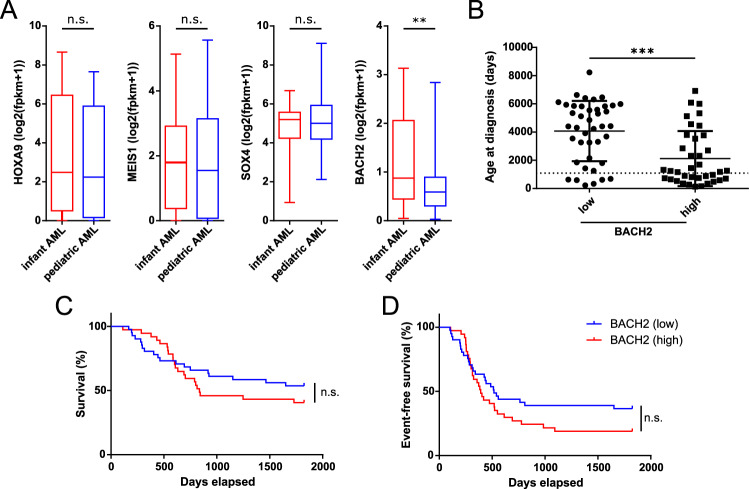
Increased *BACH2* expression in infant AML patients. **A** Comparison of *HOXA9*, *MEIS1*, *SOX4*, and *BACH2* expression between infant (<3 years (≤1095 days); *n* = 35) and pediatric (>3 years (>1095 days); *n* = 90) AML patients from the TARGET-AML study. **B**–**D** Comparison of patient characteristics stratified according to patients with the 25% highest (*n* =  37) and 25% lowest (*n* = 41) *BACH2* expression levels from the TARGET-AML study. The data were corrected for unique patient IDs, and used to depict the (**B**) age distributions, (**C**) survival curve, and (**D**) event-free survival. Data in **A** and **B** were subjected to Mann–Whitney *U* test with **p ≤ 0.01 and ***p ≤ 0.001. Data in **C**, **D** are graphed out for the first 5 years after diagnosis, and significance was tested with Log-rank (Mantel–Cox) test. n.s. not significant.

To translate our findings to human AML, we investigated the expression of *HOXA9*, *MEIS1*, *SOX4,* and *BACH2* in infant AML patients from the TARGET-AML cohort. This group of patients had previously been linked to the upregulation of a B lymphoid transcriptional program irrespective of leukemia subtype [37]. Our analysis confirmed that only *BACH2* was significantly upregulated in younger patients (Fig. 7A). Regardless, shRNA knockdown experiments with validated constructs against *SOX4* (three constructs) and *BACH2* (one construct) did not yield consistent results in human AML cell lines with recombined MLL locus (MV-4-11, THP-1, and NOMO-1) (Fig. S7A, C and data not shown).

When stratifying TARGET-AML patients according to the 25% highest and lowest *BACH2* levels, expectedly, the age of diagnosis (mean 2121 days ± 1947 vs. 4075 days ± 2137) was significantly lower for the high-expressing group (Fig. 7B). These patients also showed a trend towards inferior 5-year survival and event-free survival (median low: 517 days vs. high 392 days), although differences between the groups did not reach significance (Fig. 7C, D).

Together, these data support the concept of *BACH2* being important for the establishment of a B lymphoid transcriptional bias in infant AML and a trend towards harder to treat disease entities.

## Discussion

Our studies provide insights into the phenotypic plasticity and hierarchical organization of the LSC compartment in the prototypical H9M AML model. In contrast to previous studies, we provide evidence that lineage infidelity-displaying LSC locate at the apex of the leukemic hierarchy, while LSC subpopulations with reduced plasticity tend to induce delayed secondary leukemia. Consequently, the potency of LSC to produce lymphoid subpopulations predicted rapid disease onset and aggressive behavior in serial transplantation assays.

The limited success of AML therapy is strongly coupled to the heterogeneity and plasticity of LSCs [1], and the complex organization of the H9M LSC niche represents a valuable tool to characterize these processes in greater detail [5, 21–23]. In contrast to Gibbs et al. and our previous work [5, 10], we unexpectedly detected a relatively low frequency of mice in our present FGB-marking studies that harbored a (substantial) Lym^+^ LSC subpopulation (Fig. 2). We, therefore, hypothesize that differences in the study design with regard to the utilized cell material (5-FU BM vs Lin^−^ BM) and the expression strength of the retroviral H9M vector (MSCV vs RSF91) may have influenced lineage plasticity [38–40].

To better understand the molecular foundation for the (lack of) phenotypic plasticity, we supplemented scRNA-seq data showing a pro B cell population at the apex of the leukemic differentiation trajectory with bulk RNA-seq data (Fig. 5). These transcriptional analyses identified *Sox4* and *Bach2* as potential master regulators of phenotypic conversion and growth behavior [11, 12, 14, 41]. Interestingly, neither *Sox4* nor *Bach2* alone triggered the phenotypic conversion towards B220 expression, which could nevertheless be achieved through long-term cultivation in the presence of SCF, FLT3L, IL7, and stroma, and was accompanied by the induction of *Bach2* expression and the suppression of *Cebpa* and *Cebpb* (Fig. 6G–L). This suggests an important function of the microenvironment for *Bach2*-mediated cell expansion and possibly lineage plasticity, and fits well to the reversible acquisition of stem-like features of H9M cells through stroma interactions, as well as the increased formation of MLL-AF9-dependent B cell leukemia through a neonatal microenvironment [42, 43].

Mechanistically, BACH2 forms a feedforward loop with the myeloid-instructive transcription factor C/EBPβ and thus shifts the differentiation preferences of CLP towards a B cell fate [12]. Likewise, SOX4 antagonizes C/EBPα, with which it forms a negative feedback loop to counteracts myeloid differentiation, to promote cell proliferation and to ultimately contribute to transformation [23, 44, 45]. The upregulation of *SOX4* was identified as an independent prognostic marker for poor prognosis in AML, and our in vitro experiments demonstrate the growth-impairing effect of *Sox4* suppression on H9M cells, which was independently confirmed by in vivo studies [46].

Apart from *SOX4*, *BACH2* also holds important implications for human acute leukemia as it represents one of the few conserved upregulated genes in myeloid and lymphoid disease [47]. Interestingly, infant AML harbor a *BACH2*-enriched B cell transcriptional signature (Fig. 7) [37], which suggests therapeutic efficacy of recently developed BACH2 inhibitors as well as a B ALL adapted therapy [37, 48].

The detailed deconvolution of phenotypic plasticity based on H9M AML as surrogate model for human disease thus holds promise for the identification of optimized drug targets.

## Supplementary information

Supplementary Material

Table S1

Table S2

Table S3

Table S4

Table S5

## Data Availability

The datasets generated during the current study are available from the corresponding author upon reasonable request.
