# Supplementary material for: A pro B cell population forms the apex of the leukemic hierarchy in Hoxa9/Meis1-dependent AML
Source: Leukemia. 2022 Dec 14;37(1):79–90. doi: 10.1038/s41375-022-01775-y (PMC9883166; doi:10.1038/s41375-022-01775-y)
Supplement: Supplementary file 1 — Supplementary Material [file 41375_2022_1775_MOESM1_ESM.pdf]

Supplementary Material and Methods

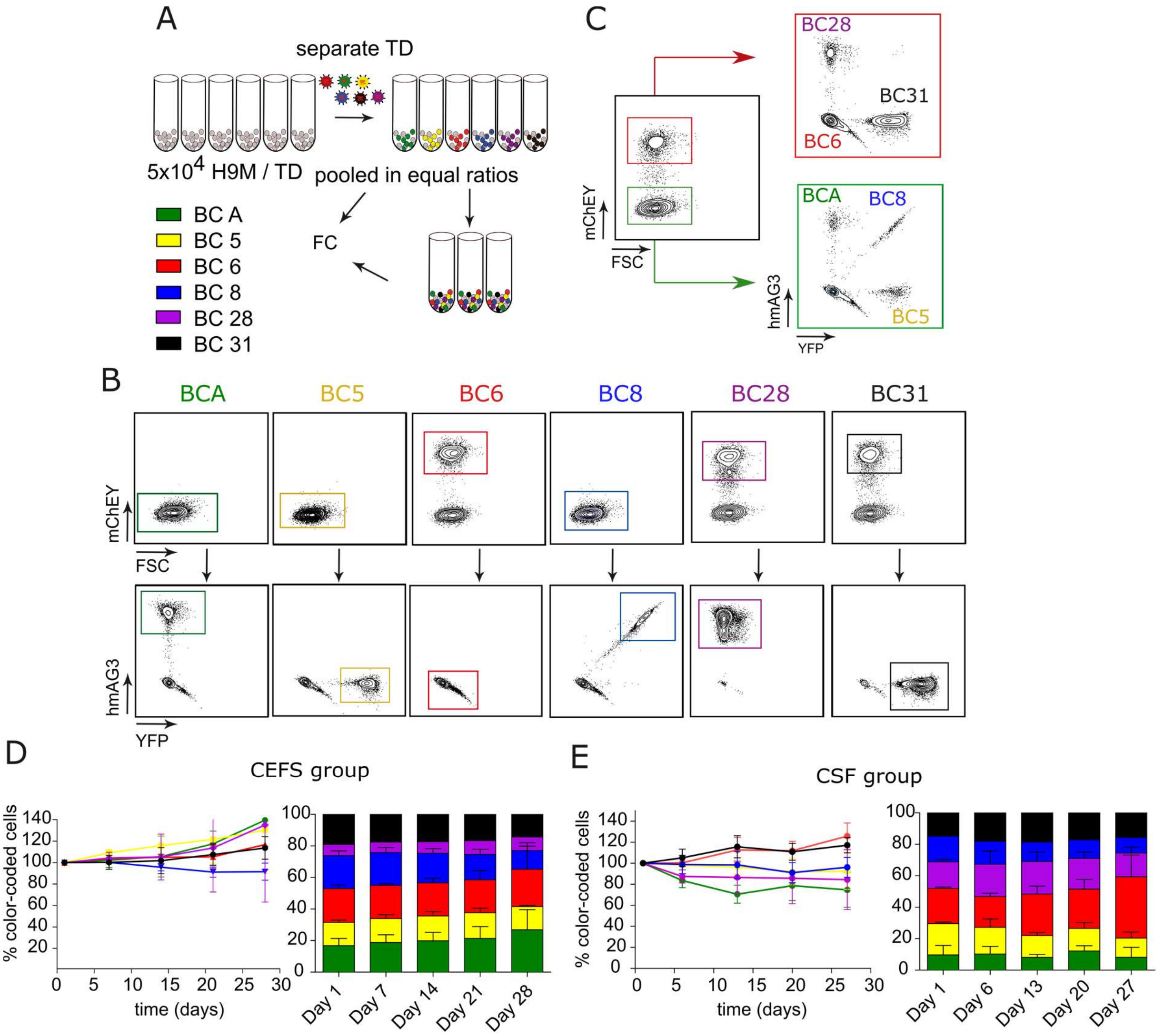

Figure S1: ***In vitro*** **validation of 6xFGB in H9M cells.**

(A) Experimental setup. H9M cells were independently transduced with each of the 6xFGB vectors carrying the CEFS or CSF promoter and expanded. Color-coded populations were maintained as separate cultures or mixed in equal ratios. Both groups were tracked over time with flow cytometry (n=3). (B) Exemplified gating strategy for single color-coded populations with the CEFS promoter. Cells were first gated on mChEY prior assessing hmAG3 and YFP expression. (C) Exemplified gating strategy for mixed color-coded populations. The gating strategy from (B) was applied. (D, E) Longitudinal tracking of single color-coded populations or mixed populations in the CEFS (D) or CSF (E) group. The different time points were normalized to the percentages of day 7. Three cell mixes from three independent transductions were tracked. TD, transduction; FC, flow cytometry. Mean ±SD is shown.

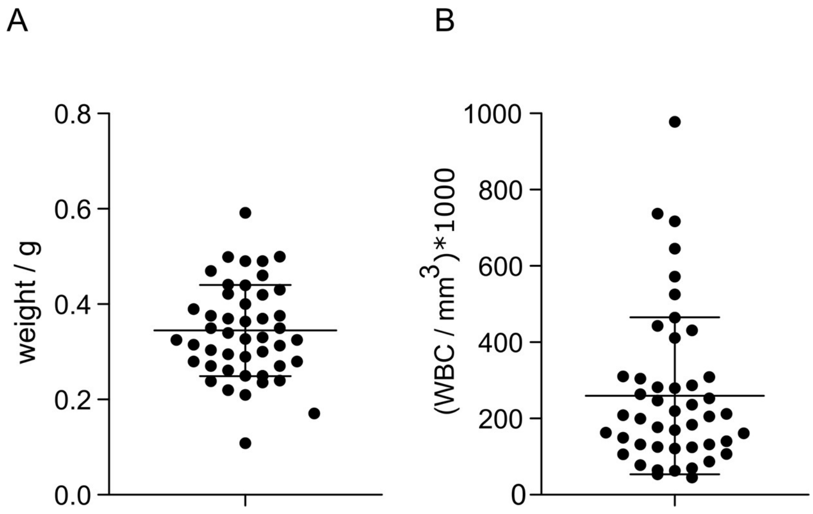

Figure S2: Disease characteristics of primary recipients.

(A) Spleen weights and (B) white blood counts (WBC) at the leukemic end-point (n=46).

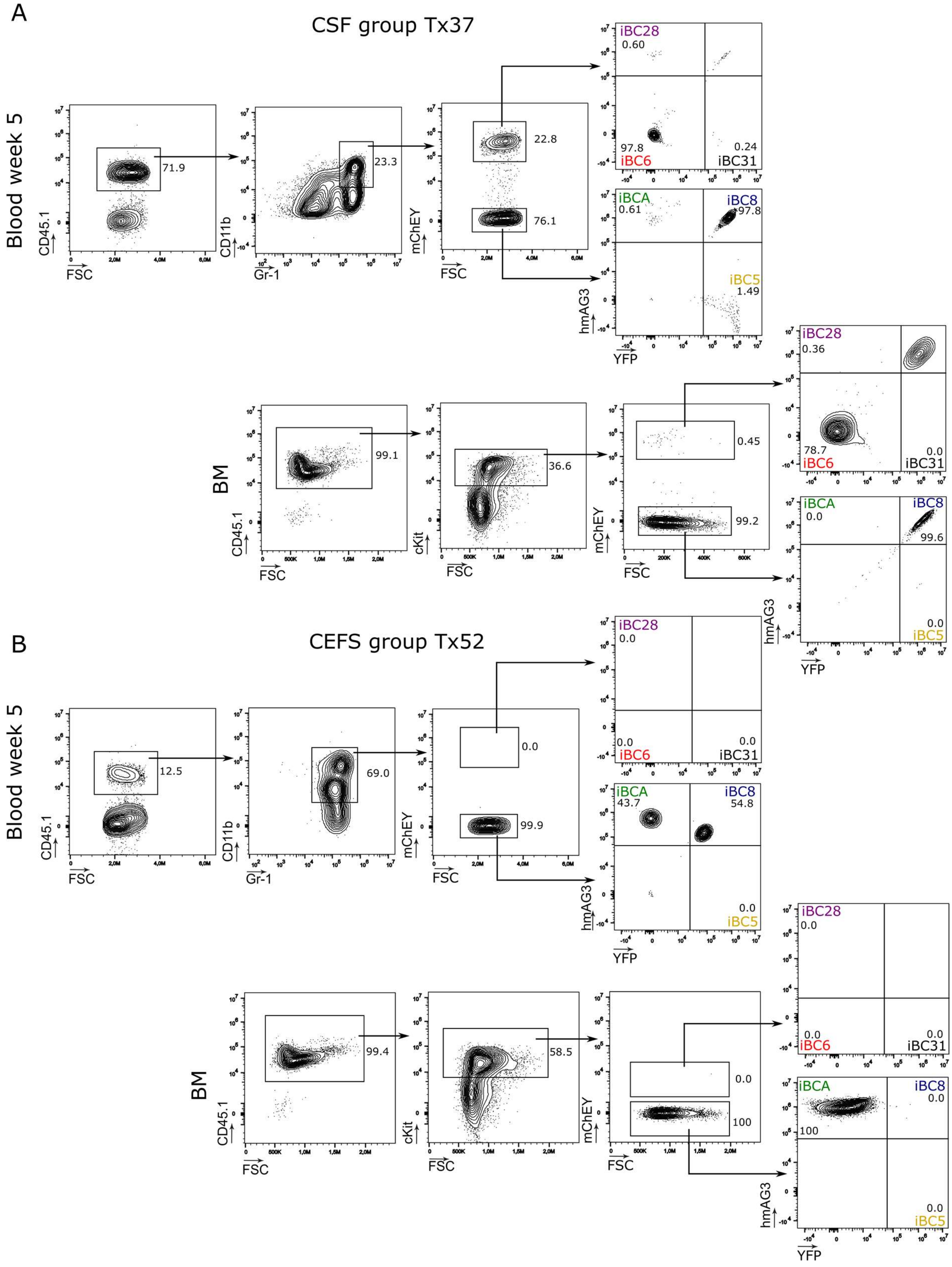

Figure S3: Gating strategy for color code identification in *in vivo* samples.

(A) Exemplified gating strategy for the identification of color codes in PB and BM of the CSF group. PB cells were first gated for the CD45.1^{+} donor population before assessing the Gr-1^{+}CD11b^{+} population and subsequent identification of all 6 color codes therein. The donor populations of the BM samples were first gated on cKit^{+} cells before assessing the color code distribution. (B) Exemplified gating strategy for the CEFS group. The same gating strategies as for (A) were applied.

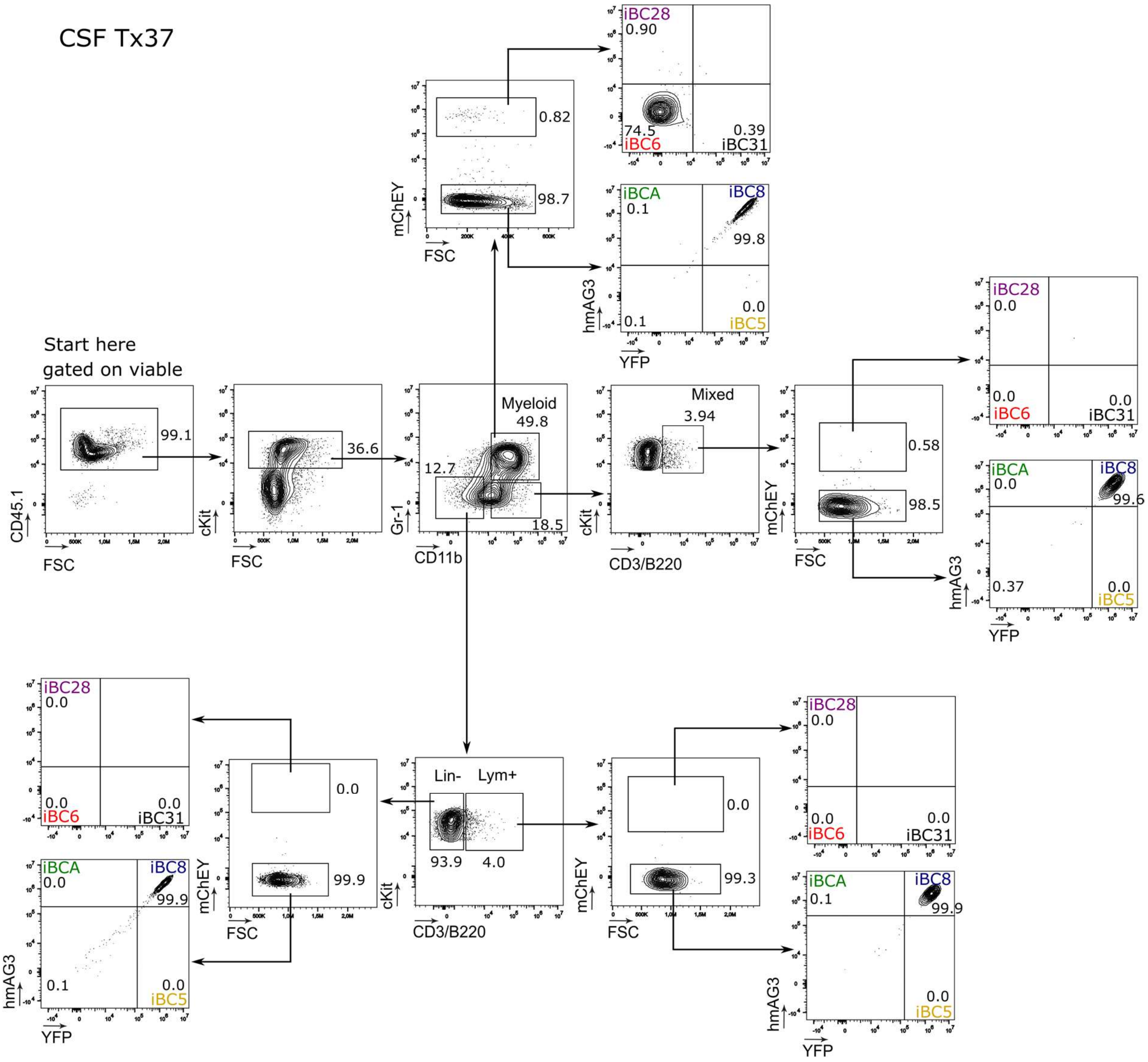
CSF Tx37
Start here
gated on viable
CD45.1
FSC
99.1
cKit
36.6
Gr-1
CD11b
Myeloid
49.8
12.7
18.5
Mixed
3.94
CD3/B220
mChEY
0.58
98.5
0.82
98.7
iBC28
0.90
74.5
iBC6
0.39
iBC31
iBCA
0.1
iBC8
99.8
hmAG3
YFP
0.0
iBC5
0.0
99.6
0.37
Lin-
Lym+
93.9
4.0
99.9
99.3

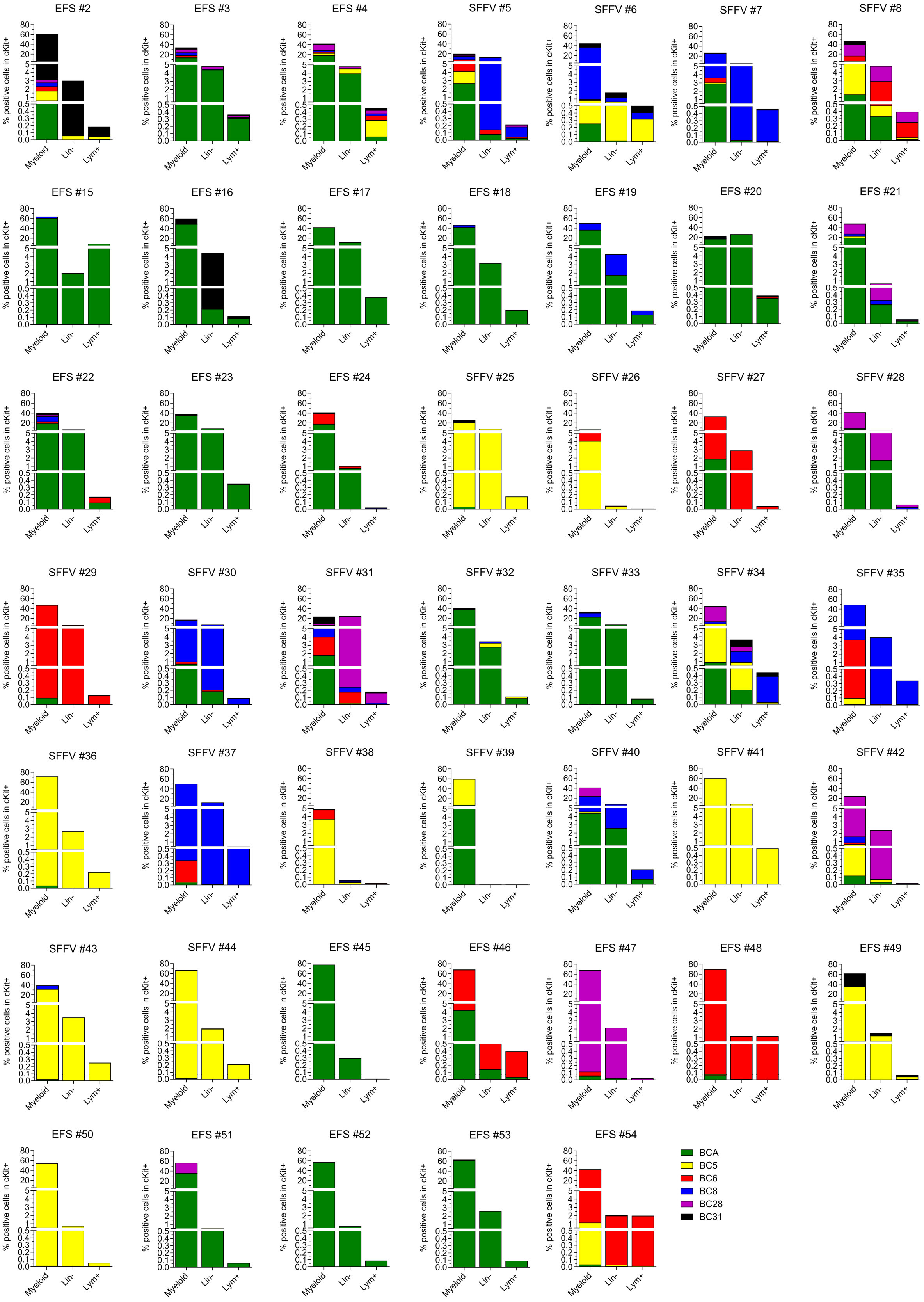

Figure S5: Summary of BM color code distributions within the LSC subpopulations from all primary recipients of H9M cells.

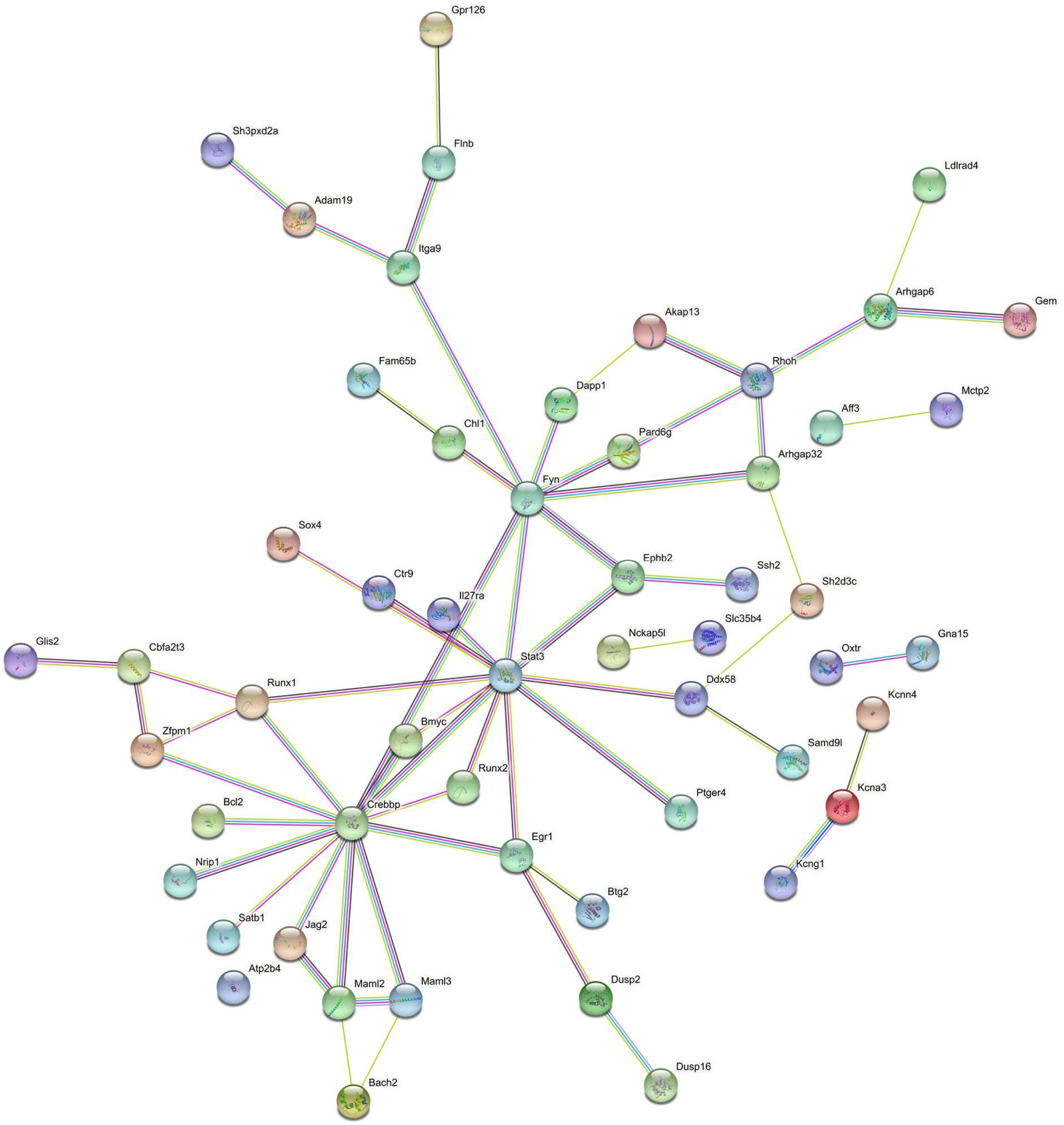

Figure S6: STRING: functional protein association networks of Lym+ LSCs.

The string analysis connects upregulated proteins in the Lym+ LSC population with P-adj<0.1. The figure was generated with https://string-db.org/.

.

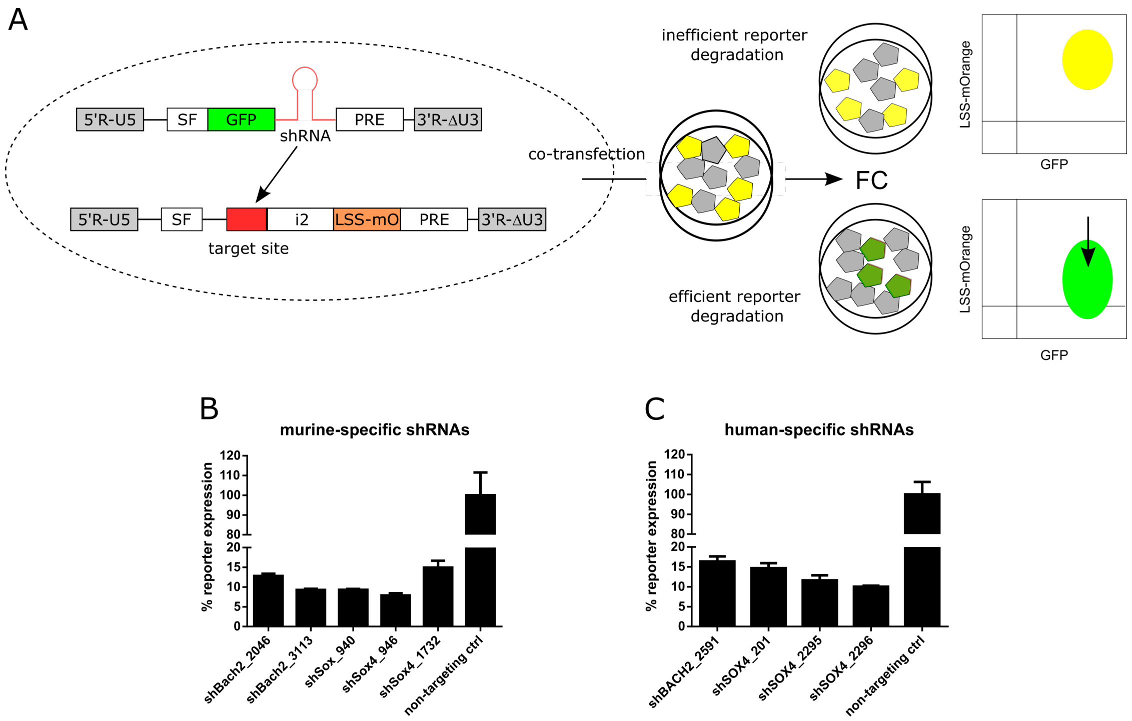

Figure S7: Transient shRNA reporter assay.

(A) Experimental design. 293T cells were transfected with the lentiviral shRNA expression plasmid as well as the lentiviral shRNA reporter plasmid (without helper plasmids). 36hrs post transfection, the cells were harvested and subjected to flow cytometric analysis. The median fluorescence intensity (MFI) of the LSS-mOrange reporter was determined in the LSS-mOrange+GFP+ double positive population and expressed as a fraction of the non-targeting control construct. Efficient shRNAs lead to a stronger loss of LSS-mOrange expression. (B-C) Characterization of (B) murine-specific shRNAs against *Bach2* and *Sox4*, and (C) human-specific shRNAs targeting *BACH2* and *SOX4*. Mean values with SD are shown for one experiment with n=3.

Supplementary Methods

Flow cytometry

For longitudinal tracking, *in vitro* cultured H9M cells were gathered in PBS supplemented with 2% FBS and 1 mM ethylenediaminetraacetic acid (EDTA, Thermo Fisher Scientific) (FACS-buffer). The color code expression was directly assessed by flow cytometry. For the flow cytometric analysis of *Bach2*, *Sox4*, *Satb1*, *Aff3*, and GFP overexpressing *in vitro* cultured H9M cells, cells were stained with cKit-PECy7 (2B8), CD11b-APCCy7 (M1/70), Gr-1-AF700 (RB6.8C5), CD3-Biotin (17A2), B220-Biotin (RA3-6B2), and Streptavidin-BV605 after blocking with CD16/32 TruStainFcX (93, all BioLegend). For the comparison of phenotypes between H9M long-term cultures in myeloid and lymphoid conditions, staining was performed with B220-PECy7 (RA3-6B2), CD115-PE/Dazzle-594 (AFS98), CD11b-APCCy7 (M1/70), CD127-PE-Cy5 (A7R34), CD135-PE (A2F10), cKit-BV785 (2B8), and Ly6C-BV510 (HK1.4) after blocking with CD16/32 TruStainFcX (all BioLegend). For the final analyses of H9M transplanted animals, aliquots of bone marrow (BM) and peripheral blood (PB) samples were thawed, blocked with CD16/32 TruStainFcXCells, and subsequently stained with CD45.1-APC (A20, eBioscience/Thermo Fisher Scientific), cKit-PECy7, CD11b-APCCy7, Gr-1-AF700, CD3-Biotin, B220-Biotin, and Streptavidin-BV605. PB samples at week 3 and 5 were stained with CD45.1-APC, cKit-PECy7, CD11b-APCCy7, and Gr-1-AF700. FACS-buffer was supplemented with 1 µM 4´,6-diamidino-2-phenylindole (DAPI, Sigma Aldrich) prior to flow cytometric analysis. Flow cytometry was performed with the CytoFlex S (Beckman Coulter, Brea, CA, USA) or the Cytek Northern Lights (Cytek Biosciences; Fremont, CA, USA) and analyzed with FlowJo 10 (BD).

H9M AML mouse transplantation assays

Starting 2 days before transplantation, CD45.2 C57BL/6J recipient mice (male and female, age 8-16 weeks) received 0.1 mg/mL Ciprofloxacin (Fresenius Kabi, Bad Homburg von der Höhe, Germany). The day before transplantation, mice were irradiated with 9 Gy using the BIOBEAM GM2000 irradiator (Eckert & Ziegler, Berlin, Germany). On the day of transplantation, sorted

H9M cells from each of the six FGB vectors were mixed in equal ratios and transplanted into the tail vein. In total, $1x10^5$ H9M cells per color code, together with $2x10^5$ radioprotective $CD45.2^+$ helper BM cells, were transplanted into each mouse. For secondary transplantations, primary H9M BM was stained with CD45.1-APC, cKit-PECy7, CD11b-APCCy7, Gr1-AF700, CD3-Biotin, B220-Biotin, and Sav-BV605. After sorting, 1000 LSCs were transplanted together with $2x10^5$ $CD45.2^+$ BM cells into $CD45.2^+$ mice. Mice were monitored on a regular basis, and maintained in a pathogen-free environment at the animal facility of Hannover Medical School. Mice were taken out of the experiment at signs of terminal disease or latest after 4 months as specified in the animal proposal. Mice were not randomized and experiments were carried out "unblinded". Sample sizes were predetermined by power analysis as part of the animal application. All mouse experiments were performed in accordance with the German animal law and approved by the Lower Saxony State Office for Consumer Protection and Food Safety (LAVES).

Sequencing data availability

Sequencing data sets are accessible through GEO accession number GSE199756.

Statistical analysis

All data and error bars are presented as the mean ± SD (standard deviation). The data points were gathered such that significant variations could be observed in the data. Differences between two groups with normal distribution were analyzed by paired Student's t test. Samples for which a normal distribution could not be determined were analyzed by Mann-Whitney U test. For groups with multiple comparisons, Kruskal-Wallis test with Dunn's multiple comparisons was applied. Survival curves were presented as Kaplan-Meier plots and analyzed for significant differences by log-rank test. *$p \leq 0.05$, **$p \leq 0.01$,***$p \leq 0.001$, and ****$p \leq 0.001$ was considered statistically significant. Due to the nature of the tests, similarities of variance were not required. Statistical tests are specified in the figure legends.

For the RNA expression data, a P-adj <0.05 was considered significant. For GSEA, a FDR<0.25 was considered to be significant. Statistical analyses were performed with GraphPad Prism (GraphPad Inc., San Diego, CA, USA). Applied statistical tests are indicated in the figure legends.

Generation of H9M cells and cultivation of cell lines

H9M cells were generated by transducing B6.SJL-Ptprc^{a}Pep3^{b}/BoyJ (CD45.1) lineage depleted bone marrow with VSVg pseudotyped concentrated pRSF91.Meis1-2A-Hoxa9.i2.Puro.LVpre gammaretroviral particles as described previously [1]. H9M cells were cultivated in 36SF medium consisting of high-glucose Dulbecco's modified Eagle medium (DMEM; Gibco/Thermo Fisher Scientific, Waltham, MA) supplemented with 15% heat-inactivated fetal bovine serum (FBS; PAN Biotech, Aidenbach, Germany), 100 U/mL penicillin and 100 µg/mL streptomycin (Pen/Strep, PAN Biotech), 0.1 mg/mL sodium pyruvate (PAN Biotech), 6 ng/mL mouse interleukin 3 (mIL3), 10 ng/mL human interleukin 6 (hIL6) and 20-100 ng/mL murine stem cell factor (mSCF) (all Peprotech, Hamburg, Germany). The cultivation of H9M cells under lymphoid cultivation conditions was performed in co-culture with OP9 cells and DMEM supplemented with 15% heat-inactivated FBS, 100 U/mL penicillin and 100 µg/mL streptomycin, 0.1 mg/mL sodium pyruvate, 20 ng/mL mSCF, 10 ng/mL human FMS tyrosine kinase 3 ligand (hFLT3L) and 5 ng/mL human interleukin 7 (hIL7, all Peprotech). OP9 bulk cultures were cultivated in Alpha Minimum Essential Medium (α-MEM) supplemented with 20% heat-inactivated FBS, 100 U/mL penicillin and 100 µg/mL streptomycin.

Lentiviral vector cloning, production, and titration

The improved 6xFGB vector system has been described before [2]. For the overexpression of cDNAs, a lentiviral expression vector was used (Addgene plasmid #171174). The dTomato cassette was exchanged by GFP, and candidate genes were amplified from H9M cells. shRNAs were selected based on high-scoring predictions with the SplashRNA algorithm, and

were subsequently cloned into the lentiviral miR-N framework [3–5]. shRNA-sequences are indicated in Table S7.

For the fluorescent shRNA reporter assay, arrayed target sites for murine-specific Sox4 and Bach2 shRNAs, as well as human-specific SOX4 and BACH2 shRNAs, respectively, were cloned into a lentiviral backbone 5' of an internal-ribosome entry site (IRES) governing the expression of the LSS-mOrange fluorescent protein. Further cloning details are available on request. All lentiviral vectors were produced in in-house propagated HEK 293T cells, which were cultivated in DMEM supplemented with 10% heat-inactivated FBS, 100 U/mL penicillin and 100 µg/mL streptomycin, and 0.1 mg/mL sodium pyruvate (DMEM^{+++}). Cells were tested for mycoplasma contaminations by PCR. One day before transfection, 5x10^6 HEK 293T cells were seeded on a 10 cm culture dish. At the day of transfection, the cells received fresh DMEM^{+++} medium supplemented with 10 mM HEPES (PAN Biotech) and 25 µM chloroquine (Sigma Aldrich, Munich, Germany). All lentiviral vectors were transiently produced by calcium phosphate-mediated transfection with 6 µg vector DNA, 6 µg RSV-Rev, 9 µg pcDNA3.GP.4xCTE (lentiviral gag/pol), and 2 µg pMD.G (VSVg). The DNA mix was incubated for 16 h before the media was renewed. 24 h and 48 h later, viral supernatants were harvested and filtered (0.22 µm). For the concentration of the virus, supernatants were ultracentrifuged at 25,000 rpm in a SW32Ti rotor at 4°C for 2 h. In DMEM resuspended viral particles were stored at -80°C until further use and titrated in 32D cells as described previously [2].

Transduction and longitudinal *in vitro* tracking of color-coded H9M cells

For the lentiviral transductions of H9M cells, 5x10^4 cells were seeded per 96 U bottom well in 36SF medium supplemented with 4 µg/mL protamine sulfate. Concentrated viral supernatants were added and incubated over-night before media renewal. For the longitudinal tracking of color-coded H9M cells, cells were individually transduced with each of the 6xFGB vectors and expanded. Between 5-11 days after transduction, gene transfer rates were first assessed by flow cytometry, and the day of analysis was arbitrarily set as day 0 of the longitudinal tracking experiment. Color-coded cell mixes were generated at the first day of flow cytometric analysis.

The same color-coded cell mixes and individual color-coded cells were measured by flow cytometry every 7 days.

Single-cell RNA sequencing and analysis

For scRNA-seq, BM samples from three different H9M transplants were chosen, including primary BM from mouse #8 and BM from its serially transplanted recipients with Lin^{-} (#57) or Lym^{+} (#63) LSCs. We performed the “Cell Hashing” approach to sequence all three samples in parallel [6]. Therefore, samples were thawed and stained as described above with cKit-PECy7, CD11b-APCCy7, Gr-1-AF700, CD3-Biotin, B220-Biotin, Streptavidin-BV605, DAPI, and three hashtag antibodies (TotalSeq-A0304 Hashtag 4 for mouse #8, TotalSeq-A0305 Hashtag 5 for mouse #57, and TotalSeq-A0306 Hashtag 6 for mouse #63; all Biolegend). After staining, BM cells were sorted for 6 different populations from each mouse (cKit^{-}, cKit^{+}CD11b^{+}Gr1^{+}, cKit^{+}Gr1^{-}CD11b^{-}CD3/B220^{+}, cKit^{+}Gr1^{-}CD11b^{+}CD3/B220^{+}, cKit^{+}Gr1^{-}CD11b^{+}CD3/B220^{-} cells and cKit^{+}Gr1^{-}CD11b^{-}CD3/B220^{-}; 1x10^{3} cells each). All hash-tagged populations were pooled for the generation of the cDNA library with the 10xGenomics Chromium Single Cell Controller (10xGenomics, Pleasanton, CA, USA). The prepared cDNA libraries were sequenced using the Illumina NextSeq 550 sequencer (Illumina, San Diego, CA, USA). The filtered data set was loaded into the Rstudio Server version 1.4.1103 [7] operating on R software version 4.0.5 [8] using the “Read10X” function of the Seurat 4.0.1 package [9]. A Seurat object was created from the gene expression matrix utilizing only genes expressed with at least 300 features in at least 3 cells. The final dataset was normalized and the top 2,000 variable genes were selected with the ”vst” method within the “*FindVariableFeatures*” function. Human cell cycle genes provided by the Sureat package were converted into mouse gene names with the biomaRt package [10], scored and regressed out using the “CellCycleScoring” and “ScaleData” functions, respectively, to avoid cell cycle effects. The data was split into each hashtag and analyzed separately. Principal component analysis was executed with the variable gene set. The first 10 principal components were selected for clustering using “FindNeighbors” and “FindClusters” at a resolution of 1.0 for hastag04 and 0.9 for both

hashtag05 and hashtag06.The visualization of clusters is based on UMAP by using the "RunUMAP" function with the first 10 principal components. Cell annotation was performed with the Cluster Identity Predictor (CIPR) based on the differential gene expression matrix of each cluster and keeping the top 10% of variable genes in the provided Immunological Genome Project (ImmGen) reference dataset [11,12].For comparison, logFC Spearman was selected. Reference cell subsets related to T cells (gD-T cell, pre-T cell, T cell and Treg), ILCs (ILC-1, ILC-2 and ILC-3), and stroma were excluded. From the resulting list of cell types, only the top hit was used for cluster annotation. For the construction of the trajectories, the Monocle (version 2.18.0) [13] package was used. The cell matrix and cluster metadata were converted to a Monocle 2 compatible file for trajectory analysis. Differential gene expression analysis was first performed with monocle 2 based on the cell annotation to obtain significant genes with q-val less than 0.01. The selected geneset was used for dimension reduction via "DDRTree" method to build the trajectory before ordering cells.

Bulk-RNA sequencing and raw data processing

For bulk-RNA sequencing, we thawed and stained secondary BM from three Lym^+ and Lin^- competent transplanted animals and isolated Lin^- and Lym^+ LSCs as described above. After staining, $1x10^4$ Lym^+ ($CD45.1^+cKit^+CD11b^-Gr\text{-}1^-B220/CD3^+$) and Lin^- ($CD45.1^+cKit^+CD11b^-Gr\text{-}1^-B220/CD3^-$) LSCs were sorted from the respective donor mice. For RNA isolation, the Qiagen RNeasy Micro Kit (Qiagen, Hilden, Germany) was used according to the manufacturer's protocol. Library preparation and sequencing was performed as describe previously [14]. Next, data were processed and a quality control was performed. For processing, the pipeline nfcore/rnaseq (version 1.4.2), which was used at the National Genomics Infrastructure at SciLifeLab Stockholm, was applied. The murine references genome and gene annotations were applied from GENCODE.org (Mus musculus, GRCm38.p6; release M25) for data alignment. For data normalization and expression analysis, the galaxy tool (Galaxy Tool Version 2.11.40.2) was used with the default settings, except for "Output normalized counts table", "Turn off outliners replacement", "Turn off outliners filtering",

and “Turn off independent filtering”, which were all set to “True”. All generated gene lists were filtered by coding genes and sorted by decreased log-FC. Gene Set Enrichment analysis (GSEA) was performed by using the Broad GSEA software [15,16] with the permutation type set to Gene_set (1000 permutations). Before querying published gene sets related to hematopoiesis [17], the gene set collections from MSigDB (version 7.4) [15] were tested first.

Gene expression analysis of human AML

To investigate *HOXA9, MEIS1, SOX4* and *BACH2* expression in human AML, the UCSC Xena Browser was used [18]. Within the GDC TARGET-AML dataset, samples with gene expression data were selected based on unique patient IDs (patients with multiple entries were excluded) and age at diagnosis (≤ 1095 days; 3 years) to generate the infant AML (n=35) subsample for comparison against all other samples (pediatric AML; n=90). Likewise, all patients were subsequently quantile stratified according to *BACH2* expression, filtered for single entries (high, n=37; low, n=41), and plotted for “Age at diagnosis”, “survival” and “event-free survival”.

Real-Time Quantitative Reverse Transcription PCR (qRT-PCR)

For the quantification of *Sox4, Bach2, Cebpa*, and *Cebpb* expression in H9M cells, qRT-PCRs were performed after the harvest and extraction of RNA using the QIAGEN RNeasy Kit (QIAGEN) according to the manufacturer’s instructions. Reverse transcription was performed with the QuantiTect Reverse Transcription Kit (QIAGEN). Quantitative PCRs were carried out with the SYBR Select Master Mix (Applied Biosystems, Waltham, Massachusetts) on an Applied Biosystems 7300 Real-Time PCR System. Each sample was measured in triplicates. Relative gene expression was calculated by the ddCt method using beta-actin (*Actb*) as loading control, and normalized to a normal BM sample. All oligonucleotides were ordered from Integrated DNA Technologies (IDT, Coralville, Iowa) and are listed in Table S8. Murine Bach2 primers correspond to Origene CAT#: MP221445, and murine Sox4 primer correspond to Origene CAT#: MP216221.

Fluorescent shRNA-reporter assay

GFP encoding lentiviral shRNA expressing vectors were cotransfected together with the appropriate lentiviral reporter construct into 293T cells (without the addition of helper plasmids) by calcium phosphate method. The cells were washed after 16 hrs, and were used for the flow cytometric assessment of LSS-mOrange reporter expression 24 hrs later. A non-targeting GFP vector was used to determine maximal expression intensity of the reporter. shRNA activity was calculated by dividing the median fluorescence intensity of the targeted reporter by the non-targeted reporter, and was subsequently expressed as a percentage of the maximal expression.

Supplementary Tables

Table S1: Summary of donor and recipient mice for LSC characterization

Table S2: hash04_Gene expression matrix-12-clusters

Table S3: hash05_Gene expression matrix-12-clusters

Table S4: hash06_Gene expression matrix-12-clusters

Table S5: Cluster annotations by CIPR

Tables S1-S5 are provided as separate data files.

Table S6: List of antibodies for flow cytometric analyses and scRNA-seq

**Surface Receptor/Reagents**	**Conjugate / Barcode sequence**	**Catalog No.**	**Supplier**
CD45.1	APC	110713	Biolegend
cKit (CD117)	PE-Cy7	105813	Biolegend
cKit (CD117)	BV785	105841	Biolegend
CD11b	APC-Cy7	101225	Biolegend
Gr1	AF700	108421	Biolegend
CD3	biotin	100243	Biolegend
B220	biotin	103203	Biolegend
B220	PE-Cy7	103221	Biolegend
Sav	BV605	405229	Biolegend
CD115	PE/Dazzle-594	135527	Biolegend
CD127	PE-Cy5	135015	Biolegend
CD135	PE	135305	Biolegend
Ly6C	BV510	128033	Biolegend
CD16/32	n.a.	101319	Biolegend
TotalSeq-A0304	AAAGCATTCTTCACG	155807	Biolegend
TotalSeq-A0305	CTTTGTCTTTGTGAG	155809	Biolegend
TotalSeq-A0306	TATGCTGCCACGGTA	155811	Biolegend

Table S7: List of cloned shRNAs

Name and splashRNA score	Sequence (5' to 3')	Vendor
mBach2_12014_2046 Score: 1.808881	TGCTGTTGACAGTGAGCGCCCAGATGATGATCAA AATGCATAGTGAAGCCACAGATGTATGCATTTTGA TCATCATCTGGATGCCTACTGCCTCGGA	IDT
mBach2_12014_3113 Score: 1.882441	TGCTGTTGACAGTGAGCGCACTGTGTACATAGTA TATATATAGTGAAGCCACAGATGTATATATATACTA TGTACACAGTATGCCTACTGCCTCGGA	IDT
mSox4_20677_940 Score: 1.1942184	TGCTGTTGACAGTGAGCGATGGGGTCTGTGCAAA AATAAATAGTGAAGCCACAGATGTATTTATTTTTG CACAGACCCCAGTGCCTACTGCCTCGGA	IDT
mSox4_20677_946 Score: 1.0526486	TGCTGTTGACAGTGAGCGCCTGTGCAAAAATAAA GAATTATAGTGAAGCCACAGATGTATAATTCTTTA TTTTTGCACAGATGCCTACTGCCTCGGA	IDT
mSox4_20677_1732 Score: 1.0882131	TGCTGTTGACAGTGAGCGCCCCTGCCGACAAGAA AGTGAATAGTGAAGCCACAGATGTATTCACTTTCT TGTCGGCAGGGTTGCCTACTGCCTCGGA	IDT
hSOX4_6659_201 Score: 1.5688812	TGCTGTTGACAGTGAGCGCTCCTACCTTGCAACA AAATAATAGTGAAGCCACAGATGTATTATTTTGTT GCAAGGTAGGAATGCCTACTGCCTCGGA	IDT
hSOX4_6659_2296 Score: 1.3925562	TGCTGTTGACAGTGAGCGAAGACGAAGAGTTTAA AGAGAATAGTGAAGCCACAGATGTATTCTCTTTAA ACTCTTCGTCTGTGCCTACTGCCTCGGA	IDT
hSOX4_6659_2295 Score: 1.1074015	TGCTGTTGACAGTGAGCGCCAGACGAAGAGTTTA AAGAGATAGTGAAGCCACAGATGTATCTCTTTAAA CTCTTCGTCTGTTGCCTACTGCCTCGGA	IDT
hBACH2_60468_2591 Score: 1.99389	TGCTGTTGACAGTGAGCGCCCAGATGATGATTAA AATGCATAGTGAAGCCACAGATGTATGCATTTTAA TCATCATCTGGATGCCTACTGCCTCGGA	IDT

*m, murine; h, human

Table S8: List of oligonucleotides for qRT-PCR

Primer name	Sequence (5' to 3')	Vendor
Bach2_FW	GTCGAAAGAGGAAGCTGGACTG	IDT
Bach2_RV	GAGGCAGGAAAAGTTGTCCAGG	IDT
Sox4_FW	GATCTCCAAGCGGCTAGGCAAA	IDT
Sox4_RV	GTAGTCAGCCATGTGCTTGAGG	IDT
Cebpa_FW	GTAACCTTGTGCCTTGGATACT	IDT
Cebpa_RV	GGAAGCAGGAATCCTCCAAATA	IDT
Cebpb_FW	CTTGATGCAATCCGGATCAAAC	IDT
Cebpb_RV	CCCGCAGGAACATCTTTAAGT	IDT
Actb_FW	CCTCCCTGGAGAAGAGCTA	IDT
Actb_RV	TCCATACCCAAGAAGGAAGG	IDT

Supplementary References

1. Hassan JJ, Lieske A, Dörpmund N, Klatt D, Hoffmann D, Kleppa MJ, et al. A Multiplex CRISPR-Screen Identifies PLA2G4A as Prognostic Marker and Druggable Target for HOXA9 and MEIS1 Dependent AML. Int J Mol Sci. 2021;22:9411.

2. Lieske A, Ha TC, Schambach A, Maetzig T. An Improved Lentiviral Fluorescent Genetic Barcoding Approach Distinguishes Hematopoietic Stem Cell Properties in Multiplexed In Vivo Experiments. Hum Gene Ther. 2021 Oct 1;32(19–20):1280–94.

3. Pelossof R, Fairchild L, Huang CH, Widmer C, Sreedharan VT, Sinha N, et al. Prediction of potent shRNAs with a sequential classification algorithm. Nat Biotechnol. 2017 Apr;35(4):350–3.

4. Adams FF, Heckl D, Hoffmann T, Talbot SR, Kloos A, Thol F, et al. An optimized lentiviral vector system for conditional RNAi and efficient cloning of microRNA embedded short hairpin RNA libraries. Biomaterials. 2017 Sep;139:102–15.

5. Adams FF, Hoffmann T, Zuber J, Heckl D, Schambach A, Schwarzer A. Pooled Generation of Lentiviral Tetracycline-Regulated microRNA Embedded Short Hairpin RNA Libraries. Hum Gene Ther Methods. 2018 Feb;29(1):16–29.

6. Stoeckius M, Zheng S, Houck-Loomis B, Hao S, Yeung BZ, Mauck WM, et al. Cell Hashing with barcoded antibodies enables multiplexing and doublet detection for single cell genomics. Genome Biol. 2018 Dec;19(1):224.

7. RStudio Team. RStudio: Integrated Development Environment for R. RStudio, PBC, Boston, MA [Internet]. 2021. 2021 [cited 2021 Oct 20]. p. 2021. Available from: https://www.rstudio.com/

8. R Core Team. R: A language and environment for statistical computing. R Foundation for Statistical Computing, Vienna, Austria. [Internet]. 2020. 2020 [cited 2021 Oct 20]. p. 2020. Available from: https://www.r-project.org/

9. Hao Y, Hao S, Andersen-Nissen E, Mauck WM, Zheng S, Butler A, et al. Integrated analysis of multimodal single-cell data. Cell. 2021 Jun;184(13):3573-3587.e29.

10. Durinck S, Spellman PT, Birney E, Huber W. Mapping identifiers for the integration of genomic datasets with the R/Bioconductor package biomaRt. Nat Protoc. 2009 Aug;4(8):1184–91.

11. Aran D, Looney AP, Liu L, Wu E, Fong V, Hsu A, et al. Reference-based analysis of lung single-cell sequencing reveals a transitional profibrotic macrophage. Nat Immunol. 2019 Feb;20(2):163–72.

12. Ekiz HA, Conley CJ, Stephens WZ, O'Connell RM. CIPR: a web-based R/shiny app and R package to annotate cell clusters in single cell RNA sequencing experiments. BMC Bioinformatics. 2020 Dec;21(1):191.

13. Qiu X, Mao Q, Tang Y, Wang L, Chawla R, Pliner HA, et al. Reversed graph embedding resolves complex single-cell trajectories. Nat Methods. 2017 Oct;14(10):979–82.

14. Lange L, Hoffmann D, Schwarzer A, Ha TC, Philipp F, Lenz D, et al. Inducible Forward Programming of Human Pluripotent Stem Cells to Hemato-endothelial Progenitor Cells with Hematopoietic Progenitor Potential. Stem Cell Rep. 2020 Jan;14(1):122–37.

15. Subramanian A, Tamayo P, Mootha VK, Mukherjee S, Ebert BL, Gillette MA, et al. Gene set enrichment analysis: A knowledge-based approach for interpreting genome-wide expression profiles. Proc Natl Acad Sci. 2005 Oct 25;102(43):15545–50.

16. Mootha VK, Lindgren CM, Eriksson KF, Subramanian A, Sihag S, Lehar J, et al. PGC-1α-responsive genes involved in oxidative phosphorylation are coordinately downregulated in human diabetes. Nat Genet. 2003 Jul;34(3):267–73.

17. Schwarzer A, Emmrich S, Schmidt F, Beck D, Ng M, Reimer C, et al. The non-coding RNA landscape of human hematopoiesis and leukemia. Nat Commun. 2017 Aug 9;8(1):218.

18. Goldman MJ, Craft B, Hastie M, Repečka K, McDade F, Kamath A, et al. Visualizing and interpreting cancer genomics data via the Xena platform. Nat Biotechnol. 2020 Jun;38(6):675–8.
