# Supplementary material for: A pro B cell population forms the apex of the leukemic hierarchy in Hoxa9/Meis1-dependent AML
Source: Leukemia. 2022 Dec 14;37(1):79–90. doi: 10.1038/s41375-022-01775-y (PMC9883166; doi:10.1038/s41375-022-01775-y)
Supplement: Supplementary file 2 — Table S1 [file 41375_2022_1775_MOESM2_ESM.pdf]

Supplementary Table S1: Summary of donor and recipient mice for functional LSC characterization.

Donor mouse	Donor population and color code	Mouse Number	included in survival curve	analyzed for LSC phenotypes	comment
#7 (SFFV)	Lin- competent BC8	55	yes	yes	
	Lym+ competent BC8	61	yes	yes	
#3 (EFS)	Lin- competent BCA	56	yes	yes	
	Lym+ competent BCA	62	yes	yes	
#8 (SFFV)	Lin- competent BC6	57	yes	yes	
	Lym+ competent BC6	63	yes	yes	
#37 (SFFV)	Lin- competent BC8	85	yes	yes	
	Lym+ competent BC8	89	yes	yes	
#35 (SFFV)	Lin- competent BC8	86	yes	yes	
	Lym+ competent BC8	90	yes	yes	
#43 (SFFV)	Lin- competent BC5	87	yes	yes	
	Lym+ competent BC5	91	yes	yes	
#17 (EFS)	Lin- competent BCA	88	yes	yes	
	Lym+ competent BCA	92	yes	yes	
#28 (SFFV)	Lin- restricted BCA	58	yes	yes	
#42 (SFFV)	Lin- restricted BC28	82	yes	yes	
#47 (EFS)	Lin- restricted BC28	83	yes	yes[1]	pre-leukemic at mandatory point of sacrifice (4M post Tx)
#33 (SFFV)	Lin- restricted BCA	84	yes	yes	
#34 (SFFV)	Lin- restricted BC28	59	yes	no	no chimerism
#22 (EFS)	Lin- restricted BC8	81	yes	no	pre-leukemic at mandatory point of sacrifice (4M post Tx)
#20 (EFS)	Mixed BCA	93	yes	yes	
#17 (EFS)	Mixed BCA	94	yes	yes	
#40 (SFFV)	Mixed BC8	98	yes	yes	
#30 (SFFV)	Mixed BC8	99	yes	yes	
#36 (SFFV)	Mixed BCA	95	yes	no	pre-leukemic at mandatory point of sacrifice (4M post Tx)
#53 (EFS)	Mixed BCA	97	yes	no	pre-leukemic at mandatory point of sacrifice (4M post Tx)

1: included in phenotypic analysis due to markedly enlarged spleen (451mg)
